# P247: Incidence of healthcare associated infections (HAIs) in maternity & children’s hospital

**DOI:** 10.1186/2047-2994-2-S1-P247

**Published:** 2013-06-20

**Authors:** M Abdelnasser

**Affiliations:** 1 Microbiology, Faculty of Medicine, Al-Azhar University, Cairo, Egypt

## Introduction

Surveillance of healthcare associated infections (HAIs) is an important step in detecting the pattern of infection prevalent in any hospital.

## Objectives

The following study aimed at analyzing the HAIs rates in a Maternity & Children’s Hospital (MCH) in relation to body sites.

## Methods

The study included 12 monthly HAIs of the Maternity & Children’s Hospital, Najran, Saudi Arabia during the year 1430 H (2009G). The total number of beds was 200 and the total number of admission was 15237 patients.

## Results

The overall annual infection rate ranged from 0.5-1.3% and the incident ratio of HAI’s ranged from 1.3-3.7/1000 patients days. The total number of patients with HAIs was 117. In this study the most common site affected with HAIs was urinary tract (10-71.4%), followed by blood (30.3-60%), surgical site (10-25%) and respiratory tract (10-25%), The most predominant microorganisms isolated from patients were *E.coli* (33/117, 28.2%), followed by Coagulase negative *Staphylococci* (27/117,23.1%),*Klebsiella pneumoniae* (18/117,15.4%), *Pseudomonas aeruginosa* (15/117,12.8%) and *Staphylococcus aureus* (7/117, 6.0 %; two of them were methicillin resistant). Other organisms as *Enterobacter cloacae*, *Enterococcus spp .*and *Candida spp*. were recovered at a rate of 2-3%. Influenza virus (H1N1) was detected in one patient. The most common sites of infection in relation to these organisms were urinary tracts (17 /32, 53.1% of patients with *E.coli*), followed by blood (26 /56, 46.4% of patients with Coagulase negative *Staphylococci*), surgical sites (4 /19, 21.0% of patients with *E.coli*).

## Conclusion

The current study stressed on the importance of surveillance in detecting the most common organism(s) causing HAIs in certain MCH. It also helps in setting up a plan for the prevention and control of these infections.

## Disclosure of interest

M. Abdelnasser Other Professor of Microbiology & Infection Control

